# Challenges and Adaptation in Otorhinolaryngology Practice During Pandemic Lockdown: Experience from a Malaysian COVID-19 Hospital

**DOI:** 10.21315/mjms2021.28.3.13

**Published:** 2021-06-30

**Authors:** Mohamed Iliyas Sultan Abdul Kader, Siti Sarah Razak, Vijayaprakas Rao Ramanna, Nor Kamaruzaman Esa, Abdul Razak Ahmad, Irfan Mohamad

**Affiliations:** 1 Department of Otorhinolaryngology-Head and Neck Surgery, Faculty of Medicine, Universiti Kebangsaan Malaysia, Cheras, Kuala Lumpur, Malaysia; 2 Department of Otorhinolaryngology-Head and Neck Surgery, Hospital Melaka, Melaka, Malaysia; 3 Department of Otorhinolaryngology-Head and Neck Surgery, Universiti Sultan Zainal Abidin, Kuala Terengganu, Terengganu, Malaysia; 4 Department of Otorhinolaryngology-Head and Neck Surgery, School of Medical Sciences, Universiti Sains Malaysia, Kubang Kerian, Kelantan, Malaysia

**Keywords:** COVID-19, Malaysia, pandemic, otorhinolaryngology, health services

## Abstract

COVID-19 has taken the world by storm: since the first few cases appeared in Wuhan, China in December 2019 and by June 2020 there were more than 10 million cases of COVID-19 cases worldwide. Malaysia had its first case in January 2020 and acted promptly by implementing several drastic measures to contain the disease. Subsequently, the Ministry of Health Malaysia has implemented guidelines and recommendations on the management of COVID-19. The Department of Otorhinolaryngology-Head and Neck Surgery (ORL-HNS) provides services for patients with ear, nose, throat, head and neck diseases and provides audiology, speech and language therapy, as well as undergraduate and postgraduate training. As the department’s staff is heavily involved in examinations and interventions of upper aerodigestive tract problems, the challenges are distinctly different from other specialties. This article discusses how COVID-19 affected ORL-HNS services and what measures were taken in Hospital Melaka, Malaysia.

## Introduction

After first reporting cases of pneumonia of unknown aetiology in Wuhan Hubei Province in December 2019 linked to a seafood and animal wet market, Chinese authorities were able to isolate the novel coronavirus on 7 January 2020 (1). The virus was renamed to ‘severe acute respiratory syndrome coronavirus 2’ (SARS-CoV-2) and its disease named coronavirus disease 2019 (COVID-19) by the World Health Organization (WHO) (2).

Due to the rapid development of the test kit reverse transcription polymerase chain reaction (RT-PCR) to detect the presence of SARS-CoV-2, countries were able to diagnose the disease, and the number of COVID-19 cases rose regionally and, by the sixth week of its appearance, COVID-19 had been detected in 20 other countries apart from China (3). On 30 January 2020, with the detection of the disease in the United States, Europe, the Middle East and Australia, WHO declared COVID-19 a Public Health Emergency of International Concern (PHEIC) (4). As of 11 March 2020, the number of cases outside China had increased 13 fold with the total number of more than 118,000 confirmed cases in 114 countries and WHO declared COVID-19 a pandemic (5). As of writing this article, a staggering 10 million cases of COVID-19 and over 500,000 deaths have occurred across the globe (6).

## COVID-19: Malaysian Scenario

The first confirmed case in Malaysia was on 25 January 2020 when three Chinese nationals who had close contact with a COVID-19-positive patient in Singapore were diagnosed as positive. This first wave of cases had 22 patients, followed by 11 days of zero cases. Subsequently, the second wave affected almost all Southeast Asian countries, with Singapore, Thailand, Indonesia and Malaysia dominating the top of the chart (7). In Malaysia, the largest cluster of cases was not only responsible for the majority of cases but also for the rise of cases in Southeast Asian countries following a missionary convention in the Kuala Lumpur suburb of Sri Petaling attended by 16,000 people known as the ‘Sri Petaling *Tabligh* cluster’ (8, 9). Since then, Malaysia has experienced some 30 clusters and sub-clusters of COVID-19 (10). As of writing this article on 1 July 2020, Malaysia has a total of 8,375 cases with 121 deaths (8).

The Malaysian government implemented multiple drastic measures to curb the infection, including the first phase of the nationwide Movement Control Order (MCO) from 18 to 31 March 2020 (11), followed by Enhanced Movement Control Order (EMCO) phases 2, 3 and 4, until 3 May 2020 (12). On 4 May, the Conditional Movement Control Order (CMCO) was established and lasted until 9 June 2020 (13), at which time the Recovery Movement Control Order (RMCO) was commenced and is still ongoing as of writing this article. The RMCO was planned to continue until 31 August 2020. With each of these phases, the government has relaxed its tight regulation, with various economic and social activities allowed in each phase (14).

### Experience of a COVID-19 Hospital

The Department of Otorhinolaryngology-Head and Neck Surgery (ORL-HNS) Hospital Melaka consists of one consultant, four specialists and 13 medical officers providing services to the 930,000-population State of Melaka in a general hospital with a total bed count of 1,091. Hospital Melaka was identified as one of the hospitals to handle COVID-19 (15). The scope of services include: i) to provide comprehensive care for patients with ear, nose, throat, head and neck diseases using up-to-date diagnostic and therapeutic tools; ii) to provide audiology services, which include screening, prevention, diagnosis and rehabilitation of hearing and peripheral vestibular disorders; iii) to provide speech and language therapy services for those with speech and language, communication, voice, feeding and swallowing disorders; and iv) to provide training for professionals and paramedics (16).

Common COVID-19 symptoms include fever, cough, runny nose and sore throat (17), all of which are common symptoms of ORL-HNS patients. The nasal cavity, nasopharynx and oropharynx are the sites of SARS-CoV-2 viral replication; even asymptomatic patients have exhibited high viral loads at these sites (18). Physical examinations of the nasal cavity, pharynx and larynx will cause discomfort and trigger pharyngeal reflex, thus inducing cough, which will result in the production of droplets or aerosols. Therefore, ORL-HNS health care workers are grouped in the high-risk exposure population (19). Hence, measures and strategies must be imposed at all levels to minimise the risk of contracting and disseminating COVID-19 while at the same time delivering essential services to patients.

### Ensuring Sustainability of Services

During MCO, all hospitals in Malaysia comprehensively adapted a number of changes according to the guidelines drafted by the Ministry of Health Malaysia (20–22). As the ORL-HNS health care workers are heavily involved in the examinations and interventions of problems of the upper aerodigestive tract, the challenges are distinctly different from other specialties (23).

## Outpatient Clinic

Before the implementation of MCO, ORL-HNS received about 500–600 patients per week during January and February 2020. Outpatient clinic procedures (endoscopy examinations of ear, nose, throat and other core outpatient procedures) were about 1,000–1,400 per month. During MCO, the number of patients was reduced by a staggering 90% with only 50–80 emergency patients per week, and procedures were reduced by 20%–30%. This was done in accordance with guidelines of the Ministry of Health Malaysia (20). Similar recommendations were made by the American Academy of Otolaryngology-Head and Neck Surgery, with the result that the majority of clinics have reduced their visits by over 80% (24).

In Wuhan, China, a group of otorhinolaryngologists managed to avoid patient to physician infection in 4,148 cases of fever and 22 cases of confirmed COVID-19 using changes to the clinic and operating theatre, as follows: i) The patient’s nasal cavity and pharynx mucosal should be well anaesthetised to reduce cough and sneeze reflexes; ii) gel-type topical anaesthetics rather than spray should be used to minimise aerosol production; and iii) the smallest diameter scope was used to minimise the likelihood of coughing and sneezing (25). We postponed approximately 2,000 patients to a later date of 4 to 6 months. In addition to this, our ORL-HNS services to cluster hospitals in Alor Gajah and Jasin were postponed during MCO. For patients with head and neck malignancy, multilevel risk assessment, multidisciplinary discussion and shared decision-making with the patients were made to constitute appropriate treatment. All these drastic measures were taken in order to provide essential services while at the same time limiting health care workers to potential COVID-19 exposure (Table 1).

To prevent shortages of personal protective equipment (PPE) in health care, non-governmental organisations, as well as prison inmates, started to produce PPE (26). Our own ORL-HNS staff started to produce PPE, such as face shields and head and shoe covers.

## Inpatient/Ward

In preparation to be fully equipped to handle the sheer volume of persons under investigation (PUI) and COVID-19 patients, a total of nine wards and one intensive care unit were emptied and converted into PUI and COVID-19 wards. This action reduced our bed capacity, and our ward became a multidisciplinary ward. All elective ward admissions (elective/day of surgery admission) were temporarily cancelled. An epidemiological history was taken from all the patients, and everyone on the ward was required to wear a mask. In-patient referrals within hospitals were screened for possible PUI and COVID-19. Discharging tendency was also high and patients were often discharged home as soon as possible following optimal treatment or transfer to a nearby health care facility for continuation of care if necessary (20). Visitors were not allowed to the wards unless required.

Inpatients with existing tracheostomy who required change, it was delayed until COVID-19 has passed (27); ORL-HNS Stanford University School of Medicine used a heat moisture exchanger (HME) instead of tracheostomy collars to avoid possible aerosolisation potential (28).

## Operating Theatre

Throughout MCO and EMCO, all our elective cases, such as tonsillectomies, sinus surgeries, mastoid surgeries, excision biopsies of lumps and bumps, were deferred. During this period, we lost a total of 240 h of operating theatre (OT) time in elective OT time and 85 h of day care OT procedure time. COVID-19 testing was done for all emergency and semi-emergency cases (airway compromise, trauma, tumours and infections), as was allowed by strict adherence to the guidelines laid by the Ministry of Health Malaysia (21, 22).

In accordance with recommendations for COVID-19 management by the Ministry of Health Malaysia, a core ORL-HNS COVID-19 team was formed comprising one specialist, two medical officers and two staff nurses, and they received formal training in wearing PPE, sample-taking and packaging from infectious disease control officers (21). Hospital Melaka has a designated operating theatre for PUI and COVID-19 patients. Anaesthetists, OT nurses, helpers and cleaners received formal training as per protocols. Tracheostomy and bronchoscopy were identified as among the high-risk procedures due to an aerosol-generating medical procedure. We performed tracheostomies and managed post-tracheostomy care according to consensus and recommendations (20, 27). To decrease the risk of aerosolisation during tracheostomy, ventilation was ceased while tracheal window is being performed and only resume ventilation after the tracheostomy cuff has been inflated. I f surgery was postponed, imaging modalities were used to monitor the progress of diseases such as tumours or cholesteatomas (29).

As of writing this article, since MCO we have managed to perform one tracheostomy for a prolonged ventilated COVID-19 patient and were called to stand by for two cases of tracheostomy in view of difficult intubation. Table 2 lists the other surgeries performed by ORL-HNS during MCO and EMCO.

## Audiology and Speech Therapy

Only emergency and selected cases that required urgent diagnosis for interventional procedures and specialised therapy were allowed during the described COVID-19 periods. General safety precautions and screening procedures were done in accordance with the guidelines (20). Audiology service was disrupted in March 2020 for a week as one of our audiologists had close contact with a COVID-19-positive patient, and all audiologists and instances of close contact had to be quarantined. Disinfection was done as per the protocol (30). Service was resumed after the audiologist’s results were deemed negative for COVID-19.

## Teaching and Training

Department of ORL-HNS, Hospital Melaka is a teaching hospital for undergraduate students, postgraduate students and provide subspecialty training in head and neck surgery. Since the implementation of MCO, all departmental teaching activities such as grand ward rounds, continuing medical education, radiology and clinical pathology conferences, mortality and morbidity meetings were cancelled and conducted via online when possible. Examinations of postgraduate students were also postponed to a later date. Postgraduate students who were initially attached to university hospitals were asked to return to public hospitals to aid in service. All of these measures affected the teaching and training of everyone involved. Similar conditions were reported in orthopaedic training in Singapore and Malaysia and urology training in Malaysia (31, 32).

## The Future Holds

We are facing the huge task of seeing all our backlogged patients during the MCO, as we have lost a total of 325 h of OT time and postponed about 2,000 outpatient appointments. Khor et al. (32) broke down the numbers and highlighted the backlogs in urology experience during COVID-19, while a similar breakdown of the backlog has been adopted to determine the numbers for ORL-HNS so that we can prepare for the work required over the next six months to catch up on 84 more outpatient consultations per week, 54 h per month for operations. These circumstances will only add difficulties to the already overburdened ORL-HNS services. Therefore, we have to be prepared to face the upcoming challenges as the practices and recommendations will continue to evolve as new evidence emerges.

## Conclusion

This unprecedented COVID-19 pandemic requires urgent public health measures and effective communication at all levels in respective departments to reduce the burden caused by COVID-19 in the form of health, social, emotional and economic disruptions. In particular, the personnel of ORL-HNS departments are at great risk of contracting SARS-CoV-2 as its consultations, clinical examinations and surgery involve the upper respiratory tract and upper aerodigestive tract, which harbour the highest viral load. Therefore, full PPE, which includes fluid-resistant FFP3/N95 masks, disposable fluid-resistant gloves and gown, googles and full-face shield, all of which should be worn at all times during patient encounters. Guidelines and recommendations are crucial and should serve as the template for practices during this pandemic. Ultimately, the standard care of the patient should not be disregarded in this time of an evolving pandemic.

## Figures and Tables

**Table 1:** Steps taken to reduce the patient load to reduce transmission in outpatient clinic

1.	Telephoning to extend the appointment with medications for long term follow-up patients and postponing blood or imaging investigation for non-urgent patients
2.	Managing only urgent cases (airway compromise, immediate post-operative patients, infections, trauma and tumours)
3.	Screening every patient using standardised questionnaires and COVID-19 risk declaration form, and temperature taken at two intervals (hospital entrance and clinic)
4.	Limiting number of visitors to the clinic by only allowing the patient and only one parent/caretaker if underage or dependent
5.	Maintaining social distancing of at least 1 metre on all encounter (appointment/registration counter, consultation). Hand sanitisers were also provided at all times
6.	Limiting number of personnel in the clinic and procedure rooms
7.	Deferring all endoscopies and microscopies unless absolutely necessary. Doctors, nurses, medical assistants should wear full personal protection equipment (PPE) during procedures (gown, N95 mask and face shield)
8.	Hospital pharmacy has encouraged all patients for drive-thru service and postal delivery medication collection services

**Table 2:** Surgeries performed during MCO, EMCO in ORL-HNS Melaka, Malaysia

	Surgery	No of surgery
1.	Diagnostic laryngoscopy and biopsy	5
2.	Foreign body removal	
	Nose (Nasoendoscopy)	9
	Throat (Esophagoscopy)	6
3.	Glossectomy (Hemiglossectomy and subtotal glossectomy)	2
4.	Incision and drainage neck abscess	6
5.	Total laryngectomy	5
	Hemilaryngectomy	1
6.	Tracheostomy	25
